# Charge-transport properties of 4-(1,2,2-tri­phenyl­vinyl)­aniline salicylaldehyde hydrazone: tight-packing induced molecular ‘hardening’

**DOI:** 10.1107/S2052252517010685

**Published:** 2017-09-01

**Authors:** Huipeng Ma, Shuo Chai, Dengyi Chen, Jin-Dou Huang

**Affiliations:** a College of Medical Laboratory Science, Dalian Medical University, Dalian 116044, People’s Republic of China; b School of Physics and Optoelectronic Technology, Dalian University of Technology, Dalian 116024, People’s Republic of China; c Key Laboratory of New Energy and Rare Earth Resource Utilization of State Ethnic Affairs Commission, School of Physics and Materials Engineering, Dalian Nationalities University, Dalian 116600, People’s Republic of China

**Keywords:** mobility, reorganization energy, packing forces, rigidity, flexible molecules, charge-transport properties

## Abstract

Based on first-principles calculations, the relationship between molecular packing and charge-transport parameters has been investigated and analysed in detail. It is found that the crystal packing forces in the flexible organic molecule 4-(1,2,2-triphenylvinyl)­aniline salicylaldehyde hydrazone (*A*) can apparently overcome the dynamic intramolecular rotations and the intramolecular steric repulsion, effectively enhancing the molecular rigidity and decreasing the internal reorganization energy. The conducting properties of *A* have also been simulated within the framework of hopping models, and the calculation results show that the intrinsic electron mobility in *A* is much higher than the corresponding intrinsic hole mobility. These theoretical investigations provide guidance for the efficient and targeted control of the molecular packing and charge-transport properties of organic small-molecule semiconductors and conjugated polymeric materials.

## Abstract 2

Taking 4-(1,2,2-triphenylvinyl)­aniline salicylaldehyde hydrazone (*A*) as an example, the relationship between molecular packing and charge-transport parameters has been computed and analysed. The conducting properties of *A* are also predicted within the framework of hopping models.

## 1. Introduction   

Charge transport in organic materials is one of the most important properties in the performance of solar cells, organic light-emitting diodes (OLEDs), organic field-effect transistors (OFETs), batteries and sensors (Root *et al.*, 2017 ▸; Shirota & Kageyama, 2007 ▸; Wang *et al.*, 2012 ▸). Since the 1950s, significant progress has been made towards improved understanding of intrinsic charge-transport phenomena in organic materials, and several models, such as the band model, the tight-binding model and the hopping model, have been proposed for the simulation and prediction of low-density intrinsic transport behaviour in organic crystals observed in OFET experiments (Grozema & Siebbeles, 2008 ▸; Shuai *et al.*, 2011 ▸).

For most organic crystal materials, the hopping model is appropriate to describe carrier transport properties, especially at room temperature, due to the fact that organic molecules are usually aggregated by weak van der Waals forces and thus the intermolecular electronic couplings are much weaker than the electron-vibration couplings for the majority of conjugated organic oligomers (Shuai *et al.*, 2014 ▸). In the hopping model, the intrinsic charge-transport rates rely mainly on two contributions. The first is the magnitude of the intermolecular electronic coupling, which is highly sensitive to the molecular packing motif, such as the relative positions of the interacting molecules and intermolecular orientations (Brédas *et al.*, 2002 ▸; Shuai *et al.*, 2014 ▸). The second is the geometric relaxation of the molecule and its surroundings when the charge carriers move. It is mostly the energy change of a single molecule on charge addition/removal, because contributions from the electronic and nuclear polarization/relaxation of the surrounding medium are significantly smaller in the solid state. According to the Marcus theory (Hush, 1958 ▸; Marcus, 1956 ▸), the rate of intermolecular electron hopping can be described by the following equation: 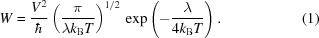
Here, *V* is the effective intermolecular electronic coupling and λ is the reorganization energy, which consists of contributions from the inner reorganization energy λ_in_ and the outer polarization λ_out_. λ_in_ is an intramolecular property that can be evaluated by quantum chemistry calculations.

In the past few years, the influence of molecular arrangement on electronic coupling has been widely studied (Brédas *et al.*, 2002 ▸; Coropceanu *et al.*, 2007 ▸; Wen *et al.*, 2010 ▸), but the relationship between molecular packing and reorganization energy has been reported only rarely so far. In general, intermolecular interactions are neglected in the evaluation of reorganization energy, and the calculated reorganization energy in the gas phase is usually adopted in the simulation of anisotropic mobility in organic materials (Deng *et al.*, 2015 ▸). Rigid molecules undergo small geometric relaxations during the charge-transfer process, and thus their calculated re­organization energy is less affected by the surroundings. With increasing molecular flexibility, however, the contribution of the intermolecular interactions to the reorganization energy may become prominent. Based on an experimental and theoretical analysis, Bunz (2010 ▸) pointed out that the crystal packing forces in oligothiophenes could apparently overcome steric repulsion and thus be helpful to the planarization of the molecular backbone. Whether or not the amplitude of the reorganization energy for a flexible molecule is also extremely sensitive to the molecular packing, just like transfer integrals, is still elusive. Monitoring of the geometric structure and reorganization energy in terms of their dependence on the molecular packing allowed valuable structure–property relationships to be established and extrapolations made to the polymer.

Recently, a new flexible organic molecule, 4-(1,2,2-triphenylvinyl)­aniline salicylaldehyde hydrazone (*A*), was synthesized by Wang *et al.* (2017 ▸), and its spectroscopic properties suggested that the conjugate plane of the phenyl rings could undergo dynamic intramolecular rotation. The high molecular chain flexibility of *A* is also supported by the weak twisting potential (see Fig. S1 in the supporting information): our quantum-chemical calculations show that the energy required to twist *A* to a 180° twist angle is only 16.5 kcal mol^−1^ (1 kcal mol^−1^ = 4.184 kJ mol^−1^). In this work, we selected flexible *A* as an example to calculate its re­organization energies in the gas phase and in the crystalline state, and to discuss the effect of packing forces on the re­organization energies. The effective electronic couplings between adjacent *A* molecules are predicted, and the way these couplings are affected by the molecular orbital shape and relative positions of the interacting units is addressed in detail. Based on these quantum-chemistry calculation results, combined with the Marcus–Hush electron-transfer theory, we simulated the anisotropic electron and hole mobilities of *A* and provide here an assessment of its field-effect properties as a potential *p*-type, *n*-type or ambipolar organic semiconducting material.

## 2. Computational results and discussion   

Firstly, we carried out density functional theory (DFT)/B3LYP calculations on the reorganization energies of *A* employing a 6-311G** basis set using the *GAUSSIAN09* program suite (Frisch *et al.*, 2009 ▸). To account for the influence of molecular packing forces on the calculated reorganization energies, the DFT simulations were performed on a molecule of *A* in the gas phase and on a supramolecular system with the molecule of *A* confined in the organic crystal (see Fig. S2*c* in the supporting information). As shown in Fig. S2(*a*), the optimized geometries of gas-phase neutral and cationic *A* deviate significantly from planarity, by 36.3 and 31.7°, respectively; in comparison, gas-phase anionic *A* is quasi-planar, showing a deviation of about 0.7°. From the large geometric relaxation associated with hole and electron transport, one can easily envisage that the electron-transfer reorganization energy (λ_e_) and hole-transfer reorganization energy (λ_h_) should be much larger than the values observed for typical π-conjugation molecules with good rigidity/planarity, such as oligoacenes and oligofurans. Our calculation results show that the λ_h_ value is 0.405 eV, which is considerably larger than those of pentacene (0.098 eV; Ma *et al.*, 2017 ▸) and sexifuran (0.238 eV; Huang *et al.*, 2011 ▸), while the λ_e_ value is 0.558 eV, *i.e.* 0.153 eV higher than the corresponding λ_h_ value. The relative magnitudes of λ_e_ and λ_h_ are consistent with the greater structural variation for an isolated molecule of *A* upon oxidation than upon reduction.

In the crystal, the neutral and charged *A* molecules are all nearly planar structures (see Fig. S2*b*): the inter-ring torsion angles lie between 8.6 and 9.9°, suggesting that the dynamic intramolecular rotations of the conjugate plane and the intramolecular steric repulsion in *A* are apparently overcome by the packing forces in the crystal. Our calculations yielded λ_h_ = 0.101 eV and λ_e_ = 0.274 eV for a molecule of *A* in a tightly packed crystal, which are much smaller than the calculation results for the gas phase. In order to evaluate the effect of the dispersion corrections on the reorganization energy results, the functional B3LYP-D3 and basis set 6-311g(d,p) were also performed for neutral and charged *A*. The calculation results show that the electron-transfer and hole-transfer reorganization energies at B3LYP-D3/6-311g(d,p) (λ_e_ = 0.273 eV and λ_h_ = 0.105 eV) nearly equal the results at B3LYP/6-311g(d,p), consistent with our previous studies (Ma & Huang, 2016 ▸). Both the structural analysis and reorganization energy calculations indicate that good solid-state packing not only maximizes the intermolecular interactions in the solid, but also enhances the molecular rigidity and decreases the internal reorganization energy effectively, especially for large and flexible conjugated molecules. Therefore, controlling the crystallization of organic compounds is an important way of improving the conducting properties of organic electronic materials.

**Figure 1:**
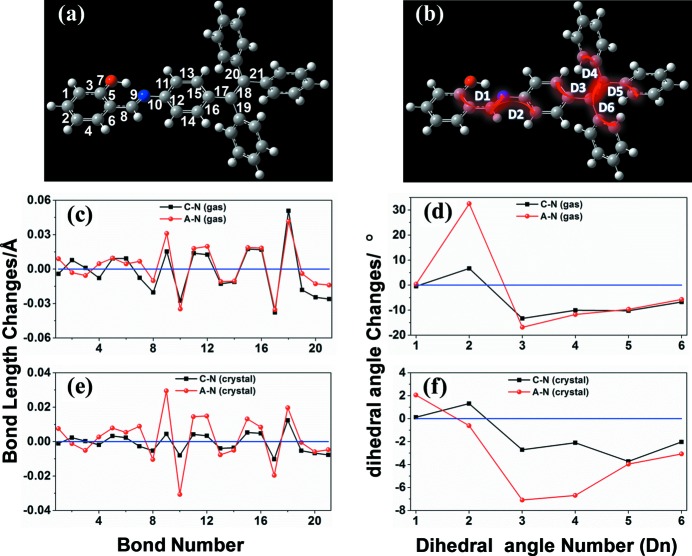
(*a*) The numbering scheme for bonds and (*b*) the numbering scheme for dihedral angles. (*c*), (*e*) Calculated variations in the bond lengths of an isolated *A* molecule in (*c*) the gas phase and (*e*) the crystal upon oxidation (black) and reduction (red). (*d*), (*f*) Calculated variations in the dihedral angles of an isolated *A* molecule in (*d*) the gas phase and (*f*) the crystal upon oxidation (black) and reduction (red).

In order to analyse qualitatively the contribution of different vibration modes to the variation in reorganization energies, the changes in bond length and dihedral angle upon oxidation and reduction of *A* are shown in Fig. 1 ▸. As shown in Figs. 1 ▸(*c*) and 1 ▸(*d*), we can see that the relaxation of the C—C bonds upon electron transfer is similar to that of the same bonds upon hole transfer, while the geometric changes in dihedral angle between the conjugate planes are more pronounced upon electron transfer than upon hole transfer, which is consistent with the observation of larger λ_e_ than λ_h_ in the gas phase. This suggests that the large difference between λ_e_ and λ_h_ (0.153 eV) comes mainly from the torsional vibration between the N atom and its neighbouring benzene ring. In the crystal, both the C—C bonds and the dihedral angles undergo geometric changes to a much smaller extent (see Figs. 1 ▸ *e* and 1 ▸ *f*), which indicates that the intramolecular C—C stretching vibrations and intramolecular rotations are greatly restricted in the aggregated state. This well explains the significantly smaller λ_e_ and λ_h_ values of *A* in the crystal than in the gas phase. Moreover, it should be noted that the geometric relaxations in the C—C bonds and dihedral angles occur predominantly upon electron transfer; in contrast, the smaller geometric changes upon hole transfer contribute less to λ_h_. Both bond length and dihedral angle variations are responsible for the larger λ_e_ than λ_h_ in the crystal.

**Figure 2:**
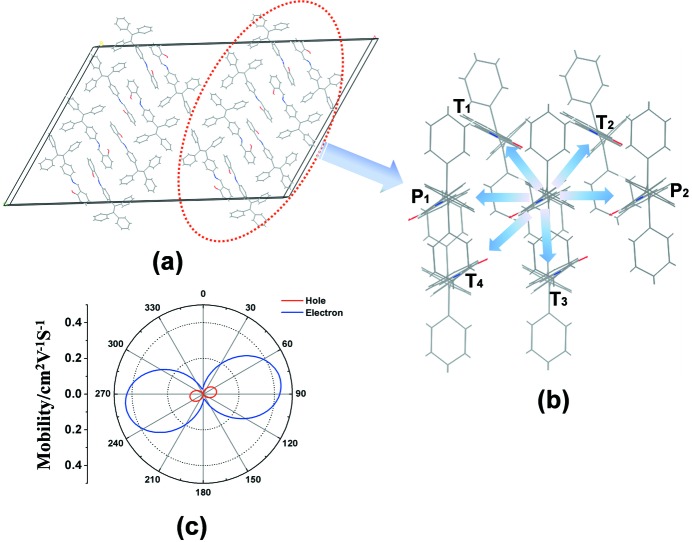
(*a*) The crystal structure of *A*, and (*b*) the hopping pathways in the same molecular stacking layers. (*c*) The calculated angle-resolved anisotropic hole-transfer mobility (red) and electron-transfer mobility (blue) of *A*; 0° corresponds to the crystallographic *b* axis.

Another significant factor governing the charge-transfer rate is the intermolecular electronic coupling. Knowledge of the relative positions of the interacting molecules is necessary to calculate the electronic coupling between adjacent *A* molecules. As shown in Fig. 2 ▸(*a*), the crystal structure of *A* is a tight herringbone arrangement, which is quite common in chain molecule crystals. Compound *A* crystallizes in the monoclinic system in space group *C*2/*c*, and each unit cell has 16 molecules (Wang *et al.*, 2017 ▸). A charge on an *A* molecule can hop to others related by translational symmetry in the same or neighbouring unit cells, as shown in pathways *P*_1_, *P*_2_, *T*_1_, *T*_2_, *T*_3_ and *T*_4_ (see Fig. 2 ▸ *b*). Examination of the geometries of the hopping pairs reveals that there are two types of molecular contact among all the pathways: (i) the two *A* molecules are parallel to each other (pathways *P*_1_ and *P*_2_), which are usually defined as *P*-type dimers; and (ii) the two *A* molecules deviate significantly from being parallel (pathways *T*_1_, *T*_2_, *T*_3_ and *T*_4_), which were defined as *T*-type dimers in our previous studies. In these hopping pathways, intermolecular electronic coupling takes the form 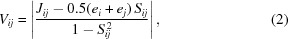
where *S_ij_* is the spatial overlap, *J_ij_* are the charge transfer integrals, and *e_i_* and *e_j_* are site energies (Coropceanu *et al.*, 2007 ▸). We carried out DFT/PW91 calculations on electronic couplings employing a TZVP basis set using the Amsterdam density functional program (*ADF*; te Velde *et al.*, 2001 ▸).

**Figure 3:**
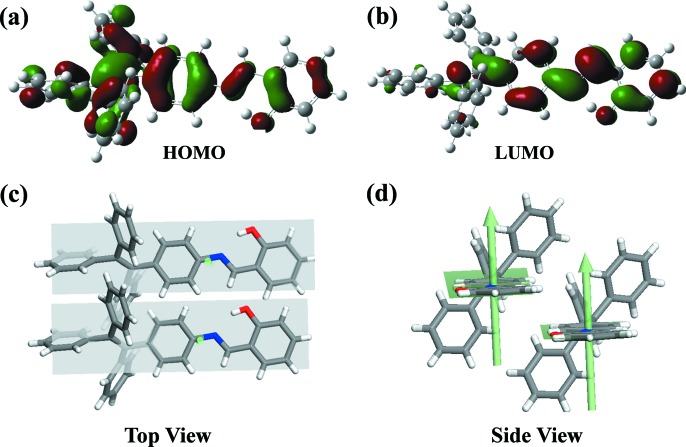
(*a*) The HOMO and (*b*) the LUMO of *A*. (*c*), (*d*) The relative displacement between two parallel *A* molecules in the *P* dimers, (*c*) top view and (*d*) side view.

The calculated electronic couplings are collected in Table 1 ▸. It can be seen that the predicted values of electronic couplings for electron transfer are about one order of magnitude larger than the corresponding ones for hole transfer. The electronic couplings for hole transport in the *P*_1_, *T*_1_, *T*_3_ and *T*_4_ dimers are 2.8, 2.9, 7.1 and 3.5 meV, respectively, whilst the corresponding electronic couplings for electron transfer are 26.8, 35.9, 48.3 and 26.3 meV, respectively. These observations can be ration­alized by an analysis of: (i) the shape of the frontier molecular orbitals of the isolated molecule of *A*; and (ii) the overlap pattern of the highest occupied molecular orbital (HOMO) or lowest unoccupied molecular orbital (LUMO) wavefunctions in the dimers, which is determined by the relative positions of the interacting units. As shown in Figs. 3 ▸(*a*) and 3 ▸(*b*), the HOMO is mainly located on intra-ring C—C bonds and partially on the C=N bond and O atom, which are aligned predominantly along the short molecular axis, whilst the LUMO is mainly located on the C—C bonds and partially on the C—N bond and O atom, which are aligned predominantly along the long axis. These distribution characteristics of the HOMOs and LUMOs lead to different extents of the spatial overlap of the HOMO–HOMO wavefunctions with the spatial overlap of the LUMO–LUMO wavefunctions in the same dimer. In the *P* dimers, there is a displacement of more than one benzene ring along the short molecular axis (see Figs. 3 ▸ *c* and 3 ▸ *d*), and thus the electronic couplings are mainly decided by the interaction between the frontier molecular orbitals distributed on the edge part of the monomers. In this configuration, the overall extent of the spatial overlap of the LUMO wavefunctions is much greater than that of the HOMO wavefunctions, which well explains the stronger electronic couplings for electron transfer than that for hole transfer. Similarly, the *T* dimers show a typical face-to-edge packing mode (see Fig. S3), and the LUMOs distributed on the edge part of the monomer are responsible for the strong electronic couplings for electron transfer.

Comparing these results, it is easy to find that the intermolecular electronic couplings are nearly one order of magnitude less than the corresponding λ. In this case, the hopping model might be preferred for the prediction of carrier mobilities. Here, we simulated the anisotropic hole and electron mobilities for *A* using the mobility orientation function proposed by Han and co-workers (Chai, Wen & Han, 2011 ▸; Chai, Wen *et al.*, 2011 ▸; Deng *et al.*, 2015 ▸; Wen *et al.*, 2009 ▸; Huang *et al.*, 2011 ▸). The estimated ranges of the hole-transfer and electron-transfer mobilities in the same molecular stacking layer are shown in Fig. 2 ▸(*c*). From the simulation results we can conclude that: (i) as a molecular crystal *A* has the potential to be developed as a potential *n*-type or ambipolar organic semiconducting material, due to the fact that the optimum values of electron-transfer mobility are larger by nearly one order of magnitude than the corresponding optimum values of hole-transfer mobility; and (ii) the maximum predicted hole-transfer and electron-transfer mobilities are present in the same direction, which means that the hole and electron mobilities could achieve their highest values at the same time if the transistor channel orientation were in the direction of Φ = 80°/170° (Φ is the orientation angle of the transistor channel relative to the crystallographic *b* axis; Φ = 0° corresponds to the crystallographic *b* axis).

## 3. Conclusions   

We have shown that the most crucial parameters affecting charge-transport properties, the charge-transfer integral and the reorganization energy, are sensitive to the molecular packing, and a densely packed crystal of the flexible π-conjugated compound *A* not only maximizes intermolecular interactions, but also enhances the molecular rigidity and decreases the internal reorganization energy effectively. Furthermore, the conducting properties of *A* were also simulated within the framework of hopping models, and our calculation results show that the intrinsic electron mobility in *A* is much higher than the corresponding intrinsic hole mobility, which means that *A* may be a better electron transporter than hole transporter.

Our theoretical investigations here are helpful for understanding the relationship between crystal structure and intermolecular charge-transport behaviours, and provide guidance for the efficient and targeted control of the molecular packing and charge-transport properties of organic small-molecule semiconductors and conjugated polymeric materials.

## Supplementary Material

Supporting information file. DOI: 10.1107/S2052252517010685/lt5007sup1.pdf

## Figures and Tables

**Table 1:** Calculated hole-transport electronic couplings *V*_hole_ (meV), electron-transport electronic couplings *V*_electron_ (meV) and intermolecular centre-to-centre distances (Å) for the different hopping pathways in the crystal structure of *A*

Pathway	Distance	*V*_hole_	*V*_electron_
*P*_1_	5.706	2.8	26.8
*P*_2_	5.706	2.8	26.8
*T*_1_	14.670	2.9	35.9
*T*_2_	14.670	2.9	35.9
*T*_3_	11.403	7.1	48.3
*T*_4_	10.752	3.5	26.3
