# Supplementary material for: Charge-transport properties of 4-(1,2,2-tri­phenyl­vinyl)­aniline salicylaldehyde hydrazone: tight-packing induced molecular ‘hardening’
Source: IUCrJ. 2017 Sep 1;4(Pt 5):695–9. doi: 10.1107/S2052252517010685 (PMC5619860; doi:10.1107/S2052252517010685)
Supplement: Supplementary file 1 [file m-04-00695-sup1.pdf]

IUCrJ

Volume 4 (2017)

Supporting information for article:

Charge-transport properties of 4-(1,2,2-triphenylvinyl)aniline salicylaldehyde hydrazone: tight-packing induced molecular `hardening'

Huipeng Ma, Shuo Chai, Dengyi Chen and Jin-Dou Huang

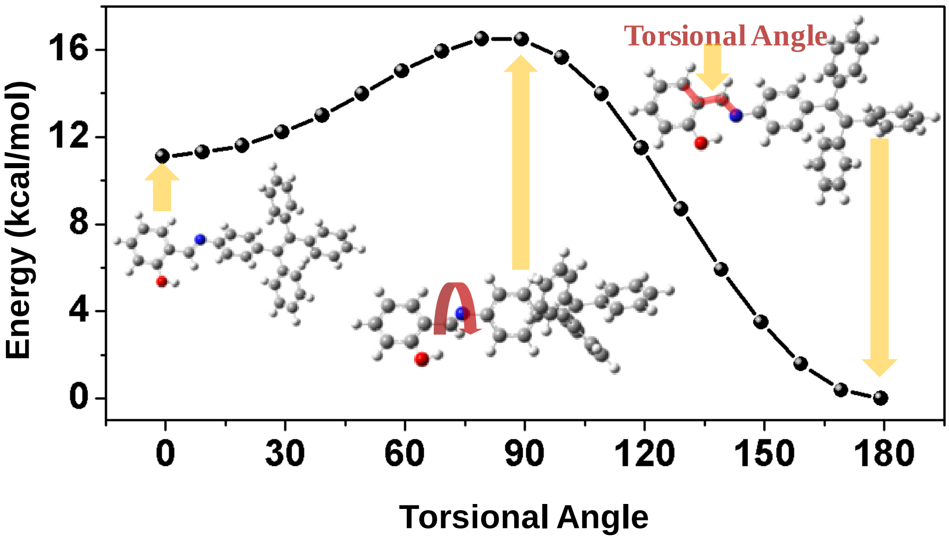

Figure S1. Variation of energy of molecule ***A*** as a function of rotation of the torsional angle between edge benzene ring and the other part of molecule ***A***.

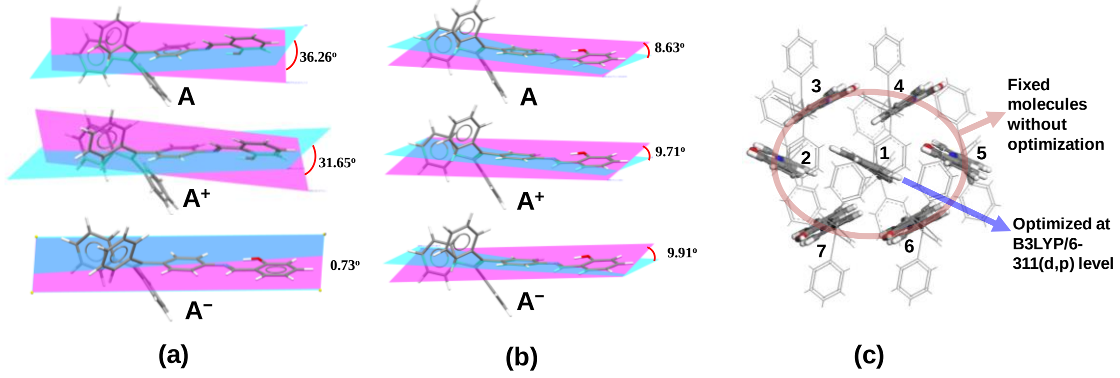

Figure S2. Dihedral angles between conjugate planes for ***A***, ***A***$^+$, and ***A***$^-$ in gas phase (a); and dihedral angles between conjugate planes for ***A***, ***A***$^+$, and ***A***$^-$ carved out of (b); (c) the computational model of the molecule ***A*** confined in the organic crystal. During structural optimization, the central molecule ***A*** (**1**) in the model is relaxed at b3lyp/6-311g(d,p), and the surrounding molecules ***A*** (**2**, **3**, **4**, **5**, **6**, and **7**) are fixed without optimization.

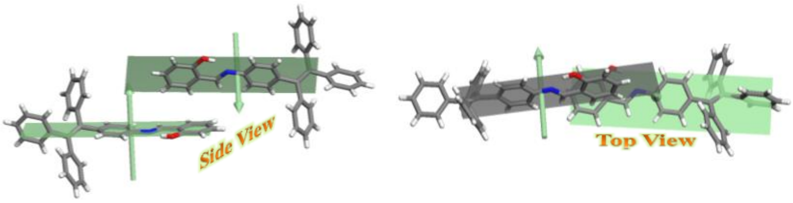

T1 dimer

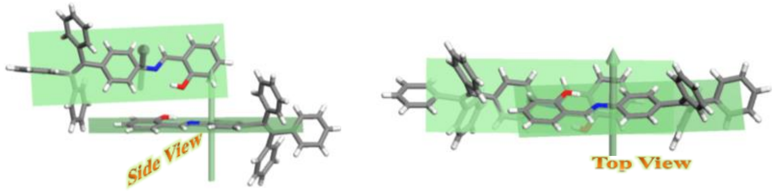

T3 dimer

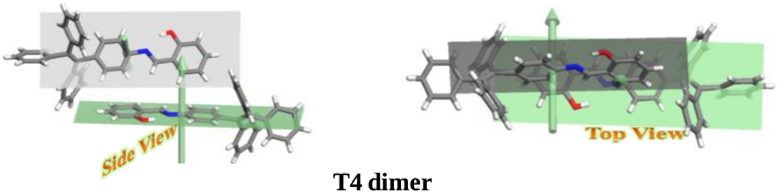

T4 dimer

Figure S3. The relative positions of two interacting ***A*** in T1 dimer, T3 dimer, and T4 dimer in top view and in side view.
